# Domestic violence and abuse related emergency room visits in Ontario, Canada

**DOI:** 10.1186/s12889-021-10501-9

**Published:** 2021-03-06

**Authors:** Sonica Singhal, Sarah Orr, Harkirat Singh, Menaka Shanmuganantha, Heather Manson

**Affiliations:** Health Promotion, Chronic Disease, and Injury Prevention Department, Public Health Ontario, 480 University Avenue, Suite 300, Toronto, ON M5G 1V2 Canada

**Keywords:** Domestic violence and abuse, Emergency room visits, Intimate partner violence, Child abuse and neglect, Elder abuse and neglect, Public health

## Abstract

**Background:**

Hospitals’ emergency rooms (ERs) are generally the first point of contact of domestic violence and abuse (DVA) victims to the health care system. For efficient management and resource allocation for ERs to manage DVA-related emergencies in Canada, it is important to quantify and assess the pattern of these visits.

**Methods:**

Aggregate DVA-related ER visits data, using relevant ICD-10-CA codes, from 2012 to 2016 were retrieved from IntelliHealth Ontario. The 2011 ON-Marg (Ontario Marginalization) indices were linked at the Dissemination Area level to ER data. Descriptive analyses including total number and rate of visits per 100,000 people were calculated, stratified by age and sex. The Slope Index of Inequality (SII) and Relative Index of Inequality (RII) were also assessed.

**Results:**

From 2012 to 2016, 10,935 (81.2% by females and 18.8% by males) DVA-related visits were made to ERs in Ontario. An annual average of 25.5 visits per 100,000 females and 6.1 visits per 100,000 males was observed. Residential instability and deprivation were significant predictors of DVA-related ER visits. No particular site of injury was indicated in 38.5% of visits, 24.7% presented with cranio-maxillofacial (CMF) trauma in isolation, 28.9% presented with non-CMF injuries, and 7.9% visits presented with both CMF and non-CMF injuries.

**Conclusion:**

This study identified that the burden of DVA-related ER visits is large enough to warrant timely public health interventions, and observed that certain populations in Ontario experience more DVA and/or are more prone to its impact. Our findings have important implications for various stakeholders involved in planning and implementing relevant policies and programs.

## Background

Domestic violence and abuse (DVA) is a public health and human rights concern that can be defined as “any form of abuse, mistreatment or neglect that a child or adult experiences from a family member, or from someone with whom they have an intimate relationship.” [1].

Victims of DVA are at an increased risk of chronic physical and mental illness, drug addiction, economic crisis, social exclusion, and further victimization [2]. In literature, terms such as “Family Violence” or “Domestic and Family Violence” are used interchangeably with DVA.

DVA can be experienced at any age. The most frequently observed forms of DVA are child abuse and neglect (CAN), intimate partner violence (IPV), and elder abuse and neglect (EAN) [2]. The Centers for Disease Control and Prevention (CDC) defines CAN as “any act or series of acts of commission or omission by a parent or other caregiver (e.g., clergy, coach, teacher) that results in harm, potential for harm, or threat of harm to a child.” [3]; IPV as “physical violence, sexual violence, stalking and psychological aggression (including coercive acts) by a current or former intimate partner.” [4]; and EAN as “an intentional act, or failure to act, by a caregiver or another person in a relationship involving an expectation of trust that causes or creates a risk of harm to an older adult (an older adult is defined as someone age 60 or older).” [5] All three forms of DVA can manifest as physical, emotional, sexual, and/or financial abuse as well as intentional or unintentional neglect [3–5].

In Canada, the General Social Survey is conducted every five years to capture information on Canadians’ experiences of victimization. The most recent cycle found that in 2014, 33% of Canadians aged 15 and older reported having experienced childhood abuse [6]. The health care system is an important point of contact, where victims of DVA can be identified and provided support. Health care professionals, specifically in emergency rooms (ERs), are often victims’ first point of contact with the health care system. This is an opportunity for health care professionals to not only treat the current DVA-related medical condition/injury, but also to provide support in mitigating the risk of its recurrence in the future through safety referral, who can provide emotional support, crisis counselling, and information and assistance with urgent moves [7, 8]. Quantifying the number and understanding the demographic characteristics of DVA-related visits made to ERs is a crucial step for estimating how much the burden of this public health issue is and where is it more concentrated [9]. Further, a better understanding of the impact of DVA-related ER visits will inform the importance of ongoing public health surveillance in the area of DVA, assessment of resource allocation required for its appropriate management and development of future preventative healthcare strategies to mitigate the burden in Canada.

Jurisdictional variation and certain indicators of marginalization (e.g. gender, ethnicity and deprivation) have been shown to increase an individual’s vulnerability to DVA [10–12]. Assessing if jurisdictional variation or levels of marginalization predict DVA-associated ER visits is crucial for directing public health and health care-related efforts aimed at mitigating inequities. Further, it is important to understand the pattern and severity of injuries sustained as a result of DVA to support ER health professionals in recognizing associated signs and symptoms. This study endeavors to fill these evidentiary gaps. To understand the overall burden and geographical distribution of DVA in the province of Ontario, our primary objective was to quantify the number of DVA-related ER visits, overall and in individual health regions of the province. To support health care professionals in understanding who bears the burden of domestic violence more, what kind of injuries are expected among DVA victims, and how severe such injuries can be, we had some secondary objectives, which included assessing the distribution of DVA-related ER visits by level of marginalization, specific injury sites involved during such visits, and disposition status of such visits (discharged, hospitalization or death).

## Methods

### Data sources

Data were extracted from two IntelliHEALTH databases, held by the Ontario’s Ministry of Health. First, the Ambulatory Visits database which contains National Ambulatory Care Reporting System (NACRS) data from the Canadian Institute for Health Information (CIHI). To understand the burden in the most recent years, all visits during calendar years 2012–2016 inclusive (i.e. January 1, 2012 to December 31, 2016) were extracted. Data were stratified by sex, age, and Local Health Integration Networks (LHINs). LHINs are crown agencies established by the Government of Ontario to provide coordinated and integrated health services at the local level [13]. Disposition status was also examined. The second database used was the Population database, specifically the “Population Estimates LHIN” dataset which contain population estimates from Statistics Canada. Population estimates were extracted for calendar years 2012–2016 inclusive and were used to calculate annual rates per 100,000 persons.

The 2011 Ontario Marginalization Index (ON-Marg), which combines various neighbourhood demographic factors into four dimensions of marginalization: material deprivation; residential instability; dependency; and ethnic concentration, was also used for this study; details of the index and these dimensions have been described elsewhere [14]. Approval for this study was secured from the Research Ethics Board of Public Health Ontario (file number 2018–003.01).

### Data extraction using ICD-10-CA codes

ER visit data were extracted from the Ambulatory Visits database using relevant International Classification of Diseases version 10 Canadian codes (ICD-10-CA codes). *Domestic violence, neglect, or abuse (DVA)*-related ER visits were determined by the presence of at least one of the following ICD-10-CA codes: maltreatment syndromes (T74); neglect and abandonment (Y06); or other maltreatment by spouse (Y07.0), parent (Y07.1), acquaintance or friend (Y07.2), other specified persons (Y07.8), or unspecified person (Y07.9). Applying these codes yielded 10,936 DVA related ER visits during 2012–2016 inclusive. One visit was excluded because patient age was not specified, resulting in a final sample of 10,935.

### Linking with 2011 ON-Marg

The ON-Marg indices were linked at the Dissemination Area level to NACRS data using the 2015 Postal Code Conversion File Plus (PCCF+; version 6C) program and associated datasets from Statistics Canada [15]. Of the sample of DVA (*n* = 10,935), 811 visits (approximately 7%) could not be linked to ON-Marg. These visits could not be linked due to: missing patient-reported postal codes in the NACRS record, no linkage between the patient-reported postal code and a Dissemination Area (DA) or living in a DA with no calculated marginalization index.

### Data analysis

Descriptive analyses were conducted. Total number of visits and rate of visits per 100,000 people were calculated stratified by age, sex, and LHIN. The Slope Index of Inequality (SII) and Relative Index of Inequality (RII) were used to assess absolute and relative inequalities in DVA-related ER visits across the distribution of each ON-Marg factor. The RII and SII are regression-based indices used to describe the socioeconomic gradient in health outcomes [16]. These measures are weighted for population size, meaning that the proportion of the total population contained in each socioeconomic group is considered in their calculation. The SII is an absolute measure of inequality that represents the slope of the regression line comparing a group’s mean health outcome (the dependent variable) to its relative rank (the independent variable). For our study, the relative rank for the regression line was calculated through assigning a cumulative proportion of the total population to each quintile for the four ON-Marg indices [16].

The RII is a relative measure of inequality that assesses the extent to which a health outcome varies across a specified measure of disadvantage [16]. Like the SII, the RII considers the size of the population and the relative disadvantage of other groups in the regression. The RII reflects the predicted value of a health outcome in the most disadvantaged divided by the corresponding value in the least disadvantaged [16]. SAS Enterprise Guide was the statistical package used for this study analysis.

## Results

### Visits by age, sex, and LHINs

From 2012 to 2016, 10,935 DVA-related visits were made to ERs in Ontario. Of these visits, 8878 (81.2%) were made by females and 2057 (18.8%) by males (Table 1). Taking population estimates into consideration, we observed an annual average of 25.5 visits per 100,000 females and 6.1 visits per 100,000 males. Broadly categorizing DVA as CAN, IPV and EAN by age stratification: 1341 (12 per 100,000) visits were made by 0–14 year olds; 8861 (21 per 100,000) by 15–59 year olds; and 733 (5 per 100,000) by 60+ year olds.

**Table 1:** Absolute numbers and annual rates (per 100,000) of Domestic Violence and Abuse (DVA) related Emergency Room (ER) visits in Ontario by age and sex, 2012–2016

Age Groups	Female		Male	
Years	N	Annual Rate (per 100,000)	N	Annual Rate (per 100,000)
0–1	60	17.2	80	21.7
1–4	222	15.8	191	12.9
5–9	180	10.0	156	8.3
10–14	306	16.9	146	7.6
15–19	1151	55.1	195	8.8
20–24	1441	60.5	215	8.7
25–29	1245	52.7	201	8.7
30–34	973	41.6	144	6.5
35–39	797	34.9	124	5.7
40–44	648	27.6	117	5.1
45–49	613	24.4	120	4.8
50–54	472	17.5	93	3.5
55–59	219	9.0	93	3.9
60–64	145	6.9	46	2.3
65–69	99	5.5	29	1.8
70–74	80	6.0	22	1.9
75–79	69	6.6	32	3.7
80+	158	9.0	53	4.8
TOTAL	**8878**	**25.5**	**2057**	**6.1**

In general, females had higher rates of DVA-related ER visits than males. This observation was consistent across all ages, except for male infants 0–1 year old who had slightly higher rates of DVA-related ER visits compared to female infants (Table 1). For females, a notable increase in rates of visits was observed starting 15–19 year age group. The rates peaked at 20–24 year age group and then showed a consistent downward trend until 65–69 year age group. Around the age of 70 years, a slight upward trend was again observed. Among males, DVA related ER visits were higher during childhood; especially, in the 0–1 year age group followed by the 1–4 year age group. After early childhood, the rates dropped and consistently remained at below 10 visits annually per 100,000.

Geographically, the trend of females making more DVA related visits than males was consistent across all LHINs in Ontario (Table 2). For both males and females, rates of DVA-related visits were highest in the North West LHIN region and lowest in the Waterloo-Wellington LHIN.

**Table 2:** Absolute numbers and annual rates (per 100,000) of Domestic Violence and Abuse (DVA) related Emergency Room (ER) visits in Ontario by LHIN^a^ and sex, 2012–2016

LHIN	Female		Male	
	N	Annual Rate	N	Annual Rate
		(per 100,000)		(per 100,000)
Waterloo Wellington	211	10.9	50	2.6
Mississauga Halton	364	11.8	105	3.5
Central West	377	16.5	89	4
Central	825	17.6	272	6
Hamilton Niagara Haldimand Brant	718	19.6	177	5
South West	500	20.3	92	3.9
Toronto Central	682	21.3	163	5.4
Central East	945	23.2	323	8.3
South East	352	28	92	7.6
Champlain	1024	30.8	146	4.5
North Simcoe Muskoka	496	41.5	142	12.1
North-East	652	45.6	147	10.5
Erie St. Clair	795	49.1	80	5.1
North-West	798	134.7	151	25.7
Total visits	**8878**	**548**	**2057**	**6.1**

^a^LHIN- Local Health Integration Network

### Visits by the level of marginalization

Table 3 displays rates (per 100,000) of DVA-related ER visits from 2012 to 2016 and also the SII and RII estimates for each ON-Marg factor.

**Table 3:** Domestic Violence and Abuse (DVA) related Emergency Room (ER) visits and inequalities index scores by marginalization indices and quintiles, 2012–2016 (*n* = 10,124)

Marginalization Indices	Annual rate per 100,000 persons (no. of cases)					Slope Index of Inequality (95% CI)	Relative Index of Inequality (95% CI)
	Q1^a^	Q2	Q3	Q4	Q5^b^		
Instability	9.4 (1354)	11.5 (1422)	15.6 (1817)	19.7 (2343)	23.4 (3188)	−18.6 (− 22.7 to −14.5) †	3.9 (2.6 to 5.7) †
Deprivation	8.7 (1010)	10.6 (1313)	13.9 (1741)	15.9 (2071)	27.8 (3989)	− 22.4 (− 38.9 to − 6.0)†	5.9 (0.7 to 47.2)
Dependency	14.2 (2415)	15.8 (2026)	17.2 (2006)	16.7 (1811)	16.2 (1866)	− 2.8 (− 7.4 to 1.9)	1.2 (0.9 to 1.6)
Ethnic Concentration	20.2 (2079)	19.0 (2035)	14.7 (1725)	12.6 (1657)	14.6 (2628)	8.2 (−2.6 to 19.1)	0.6 (0.3 to 1.2)

^a^Least marginalized, ^b^ Most marginalized, † Statistically significant (*p* < 0.05)

Residential instability and deprivation were significant predictors of DVA-related ER visits. Those in the least stable neighbourhoods (Q5) made 14.0 more (Q5:23.4, Q1:9.4) annual visits per 100,000 persons to the ERs for DVA-related issues than those in the most stable neighbourhoods (Q1). This inequality was significant both relatively (RII 3.9, 95% CI 2.6 to 5.7) and as an absolute difference (SII -18.6, 95% CI − 22.7 to − 14.5). Similarly, those who were the most deprived (Q5) made 19.1 (Q5: 27.8, Q1: 8.7) more annual visits per 100,000 persons to the ERs than the least deprived (Q1), which was a significant absolute difference (SII -22.4, 95% CI − 38.9 to − 6.0). There were no significant relationships between dependency or ethnic concentration and the rate of DVA-related ER visits.

### Injury sites involved in DVA-related ER visits

Among 10,935 DVA-related ER visits, 4215 (38.5%) did not have any physical injuries indicated; this absence was more often observed among female visits (41%) than male visits (28%). Table 4 shows distribution of DVA related ER visits by types of injuries. Of the visits where physical injuries were indicated: 2697 visits (24.7%) presented with cranio-maxillofacial (CMF) trauma in isolation; 862 (7.9%) visits presented with both CMF and non-CMF injuries; and 3161 (28.9%) visits presented only with non-CMF injuries.

**Table 4:** Distribution of Domestic Violence and Abuse (DVA) related Emergency Room (ER) visits by types of injuries, 2012–2016

Injury Type	Female		Male		Total	
	N	%	N	%	N	%
CMF^a^ only, no other defined injuries or burns/corrosions	1947	21.9%	750	36.5%	2697	24.7%
CMF plus other defined injuries or burns/corrosions	721	8.1%	141	6.9%	862	7.9%
No CMF, only other defined injuries or burns/corrosions	2571	29.0%	590	28.7%	3161	28.9%
No physical injury or burns/corrosions indicated	3639	41.0%	576	28.0%	4215	38.5%
Total VISIts related to DVA	**8878**	**100.0%**	**2057**	**100.0%**	**10,935**	**100.0%**

^a^CMF-Cranio-maxillofacial trauma

Table 5 shows injury sites indicated during DVA-related ER visits. As more than one site could be involved during a visit, the total of all number of sites involved exceeds the number of visits. The wrist and hand (16.9%), neck (15.1%), and thorax (13.9%) were most commonly affected sites outside of the CMF region.

**Table 5:** Sites of injuries associated with Domestic Violence and Abuse (DVA), 2012–2016 (*n* = 6720 visits)*

ICD-10-CA ± code	Injury site	Female	Male	TOTAL
		N (%)**	N (%)**	N (%)**
S00-S09	Cranio-maxillo facial injuries	2647 (50.5%)	883 (59.6%)	3530 (52.5%)
S10-S19	Injuries to the neck	543 (16.5%)	63 (8.6%)	606 (15.1%)
S20-S29	Injuries to the thorax	434 (13.2%)	125 (17.1%)	559 (13.9%)
S30-S39	Injuries to the abdomen, lower back, lumbar spine and pelvis	349 (10.6%)	85 (11.6%)	434 (10.8%)
S40-S49	Injuries to the shoulder and upper arm	363 (11.0%)	76 (10.4%)	439 (10.9%)
S50-S59	Injuries to the elbow and forearm	331 (10.1%)	98 (13.4%)	429 (10.7%)
S60-S69	Injuries to the wrist and hand	553 (16.8%)	127 (17.4%)	680 (16.9%)
S70-S79	Injuries to the hip and thigh	158 (4.8%)	37 (5.1%)	195 (4.8%)
S80-S89	Injuries to the knee and lower leg	250 (7.6%)	38 (5.2%)	288 (7.2%)
S90-S99	Injuries to the ankle and foot	115 (3.5%)	27 (3.7%)	142 (3.5%)
T00-T14	Injuries involving multiple body regions	929 (28.2%)	169 (23.1%)	1098 (27.3%)
T20-T32	Burns and corrosions	21 (0.6%)	7 (1.0%)	28 (0.7%)

± International Classification of Diseases – version 10-Canada. *injury sites from *n* = 6720 ER visits; 5239 by females and 1481 by males. **calculated as % of total visits for females, males, or total.

### Disposition status of DVA-related ER visits

Disposition status refers to the status of patients at the conclusion of an ER visit, which depends on the severity of the medical condition of the patient. From 2012 to 2016, fewer than five patients making DVA-related visits had disposition codes indicating that they died in the ER; therefore, that data are not presented. Hospitalization occurred in 475 visits (4.3%), with 7.1% of visits by males resulting in hospitalization compared to 3.7% of female visits.

Higher hospitalization rates tend to be observed among the youngest and the oldest age groups (Table 6). Among females, 18% of visits made by 0–1 year olds required hospitalization, after which the rates ranged from 1 to 5% until the age of 60. After the age of 60 years, the rates start trending upwards, reaching 42% among 80 year olds and above. For males, 38% of 0–1 year olds, and 12% of 1 to 4-year olds were admitted to a hospital from ER. The proportion remained low after that for most of the age groups until the age of 70, after which the proportions again raised notably (Table 6).

**Table 6:** Proportion of Domestic Violence and Abuse (DVA) related Emergency Room (ER) visits associated with hospitalization by age group and sex, 2012–2016

Age groups	Females		Males	
	DVA-related ED visits	Hospitalizations	DVA related ED visits	Hospitalization
Years	**N**	**N (%)**	**N**	**N (%)**
0–1	60	11 (18%)	80	30 (38%)
1–4	222	11 (5%)	191	22 (12%)
5–9	180	N/R	156	N/R
10–14	306	15 (5%)	146	5 (3%)
15–19	1151	21 (2%)	195	N/R
20–24	1441	16 (1%)	215	6 (3%)
25–29	1245	26 (2%)	201	N/R
30–34	973	20 (2%)	144	5 (3%)
35–39	797	13 (2%)	124	6 (5%)
40–44	648	18 (3%)	117	N/R
45–49	613	22 (4%)	120	N/R
50–54	472	10 (2%)	93	N/R
55–59	219	11 (5%)	93	N/R
60–64	145	17 (12%)	46	N/R
65–69	99	14 (14%)	29	N/R
70–74	80	13 (16%)	22	7 (32%)
75–79	69	24 (35%)	32	14 (44%)
80+	158	67 (42%)	53	22 (42%)
Total	**8878**	**329 (4%)**	**2057**	**146 (7%)**

N/R: Not reported as the count is less than 5

## Discussion

Using health administrative data, we estimated that 10,935 DVA-related ER visits were made in Ontario between 2012 and 2016. This is equivalent to approximately six DVA-related ER visits per day in Ontario. This burden is large enough to warrant timely public health interventions in ERs, including capacitating healthcare professionals to recognize clinical manifestations of DVA and make appropriate referral for their patients.

While not all DVA-related incidents will require physician attention in an ER, it is likely that the true burden of DVA-related ER visits is higher than the rates of identified cases reported in this paper. In 2015 alone, 25,929 cases of IPV (not including CAN and EAN) were reported to police in Ontario, which is an average of 71 cases per day [6]. Further, it is estimated that under one-third (31%) of victimization is reported to the police [17]. There can be various reasons for not reporting victimization including fear of retaliation, shame, stigma, and fear of discussing such a personal issue [18–20]. Child victimization is especially under-reported to police, as children may be fearful of consequences of reporting, lack social support to file a report, or may be unaware of the criminal nature of the abuse/act they experience [14]. It is also estimated that less than 20% of victims report their DVA associated injuries to their family physicians [21].

Our data shows that females made more DVA-related ER visits compared to males, which is consistent with findings from other international jurisdictions, [22–25] and also corroborates Canadian police data [6]. This trend, however, was reversed among infants 0–1 years old. Other studies have also found physical abuse to be higher among male infants [26, 27]. Importantly, irrespective of sex, the rates of DVA-related visits among children < 15 years of age are the highest for infants. Further research is needed to confirm and elucidate the factors influencing higher rates of visits among younger children.

Geographically, the highest rates of DVA-related ER visits were observed in the North West LHIN and the lowest in the Waterloo-Wellington LHIN. Census data from 2001 indicate that the North West LHIN had a higher unemployment rate and a larger proportion of residents who did not complete a high school education in comparison to Ontario overall [28]. The opposite was observed for the Waterloo Wellington region during this period [29]. Since there is evidence to suggest that these social determinants of health shape individuals’ vulnerability to DVA, this could help explain these findings to a certain extent [30–32]. Nevertheless, further explorations to understand contextual differences would be beneficial for customizing local public health interventions.

In our investigation, area-level residential instability and material deprivation were found to be associated with higher rates of DVA-related ER visits in Ontario. Although relevant empirical studies conducted in a Canadian context is limited, the association between individual and neighbourhood level housing instability and intimate partner violence has been well established in the U.S. literature [33, 34]. Many co-occurring challenges related to IPV and residential instability shape the relationship between the two [35]. For instance, there is evidence to suggest that residential instability is linked to weakened social ties, which may prevent neighbours from collectively intervening during cases of violence [36–38]. The association between individual and neighbourhood level material deprivation and DVA is also well established in the U.S. literature [25, 39–42]. As is the case for residential instability, there are likely many factors that influence the relationship between material deprivation and DVA. For instance, economic instability could shape vulnerability to DVA through various direct and indirect pathways such as: continuing in abusive relationships due to economic dependence on partners, [43] being the victim of CAN due to parental stress associated with financial hardship, [42] and experiencing increased susceptibility to EAN due to low-levels of social support (which is associated with low socioeconomic status among older adults) [44].

Approximately 4% of DVA related-ER visits resulted in hospitalization between 2012 and 2016 in Ontario. Two studies examining assault-related ER visits among adult patients observed comparable numbers. One study conducted in the U.S. found that about 5% of ER visits due to IPV resulted in hospitalization [25]. Similarly, a Denmark study found that 6% of violence-related ER visits lead to hospitalization [45]. A higher proportion of male visits than female visits resulted in hospitalization, indicating their injuries are more likely to be severe enough to warrant hospital admission. Also, among DVA-related visits, a higher proportion of males, irrespective of age, presented with CMF trauma. National and international studies examining CMF trauma in hospital departments have also observed similar results in general [46–49]. By age, higher hospitalization rates were found among younger (< 4 years) and older age groups (70+). Other studies have also observed similar trends [50, 51]. Potential explanations for this pattern include these populations being more vulnerable to the impact of injuries sustained during abusive episodes and/or more likely to delay seeking medical attention until becoming more severely injured [48, 50, 52].

A limitation of our data is that it is based on ER visits where DVA has been identified and documented. Previous studies have shown that ER visits caused by DVA go underreported by as much as 87%, suggesting that our estimates are much lower than the actual number of DVA-related ER visits [22]. One Ontario study found that one fifth of the children with abuse-related injuries had been missed during initial medical visits [53]. Although these cases were eventually detected in subsequent medical encounters, the study was unable to include cases of abusive fractures that had never been detected in medical settings, indicating that the actual number could be even higher [53]. Coding inaccuracies can be another limitation of the data available. Another limitation was that the ON-Marg used data as recent as 2011; however, since our report examines data from 2012 to 2016 any Dissemination Area level changes occurring after 2011 will not have been captured by our analysis. Also, patients’ individual socioeconomic situation or family condition were not considered in this study; variables of geographic marginalization and individual’s socioeconomic or family status could be related and/or interacted, which could not be captured in these observations. Future studies, both qualitative and quantitative, can include these dimensions to further assess factors attributing to the vulnerability of victims.

Irrespective of these limitations, this is the first Canadian study, to our knowledge, to examine the province-wide DVA-related ER visit data. Estimates from other provinces would be helpful in developing nation-wide strategy to curb this public health epidemic.

## Conclusion

DVA is an important public health and social justice issue that continues to affect the lives of many vulnerable people in our communities. We have identified that the burden of DVA related ER visits in Ontario is large enough to warrant timely public health interventions; shown that the most common site of injury is the CMF region; and observed that more marginalized populations and certain regions in Ontario experience more DVA and/or are more prone to its impact. Our findings have important implications for provincial and municipal stakeholders involved in planning and implementing relevant policies and programs, including administrators, politicians, health and public health professionals, and researchers.

## Data Availability

Public Health Ontario (PHO) cannot disclose the underlying data. Doing so would compromise individual privacy contrary to PHO’s ethical and legal obligations. Restricted access to the data may be available under conditions prescribed by the Ontario *Personal Health Information Protection Act, 2004*, the Ontario *Freedom of Information and Protection of Privacy Act*, the *Tri-Council Policy Statement: Ethical Conduct for Research Involving Humans (TCPS 2 (2018))*, and PHO privacy and ethics policies. Data are available for researchers who meet PHO’s criteria for access to confidential data. Information about PHO’s data access request process is available on-line at https://www.publichealthontario.ca/en/data-and-analysis/using-data/data-requests.

## Abbreviations

CAN: Child Abuse and Neglect
CDC: Centers for Disease Control and Prevention
CIHI: Canadian Institute for Health Information
CMF: Cranio-maxillofacial trauma
DVA: Domestic Violence and Abuse
EAN: Elderly Abuse and Neglect
ER: Emergency Room
ICD-10-CA: International Classification of Diseases Version 10 Canadian codes
IPV: Interpersonal Violence
LHIN: Local Health Integration Network
NACRS: National Ambulatory Care Reporting System
ON-Marg: Ontario Marginalization Index
RII: Relative Index of Inequality
SII: Slope Index of Inequality
